# A new species of *Amphictene* (Annelida, Pectinariidae) from the northern South China Sea

**DOI:** 10.3897/zookeys.545.6454

**Published:** 2015-12-14

**Authors:** Jinghuai Zhang, Yanjie Zhang, Jian-Wen Qiu

**Affiliations:** 1 South China Sea Environmental Monitoring Center, State Oceanic Administration, Guangzhou, P. R. China; 2 Department of Biology, Hong Kong Baptist University, Hong Kong, P. R. China

**Keywords:** polychaete, taxonomy, systematics, Guangdong

## Abstract

Pectinariids are a family of polychaetes commonly found in shallow coastal waters around the world, but their diversity is poorly known along the coasts of Asia. Here we describe *Amphictene alata* **sp. n.** (Pectinariidae), based on 15 specimens collected from the coastal waters of Guangdong in the northern South China Sea. This new species can be distinguished from all other 13 described species and one described subspecies of *Amphictene* by having a pair of dorsolateral lobes on segment 3, a pair of large lateral lobes on segment 21, and more scaphal hooks (26 to 37 pairs).

## Introduction

Pectinariids, commonly called ice cream cone worms, are morphologically unique polychaetes with a cone-shaped tube which they build from sand grains, and a set of golden opercular paleae which they use for digging into soft sediment (Hartman 1941; Wolf 1984; Hutchings 2000; Rouse and Pleijel 2001). Currently five genera (*Amphictene*, *Pectinaria*, *Cistenides*, *Lagis* and *Petta*) are recognized in the family Pectinariidae (Fauchald 1977; Wolf 1984; Hutchings and Peart 2002).

*Amphictene* can be distinguished from other genera of Pectinariidae by having a cirrate dorsal opercular rim. Currently this genus has 13 recognized species and one recognized subspecies (Hutchings and Peart 2002; García-Garza and de León-González 2014). Among them only three species have been described from Asia: *Amphictene moorei* (Annenkova, 1929) from the east coast of Russia, *Amphictene japonica* (Nilsson, 1928) from Japan and *Amphictene leioscapha* (Caullery, 1944) from Indonesia. The diversity of Pectinariidae is poorly known along the coasts of China, with only seven recorded species, some of which are South African or European species whose identities should be confirmed (Yang and Sun 1988; Sun and Qiu 2012). Here we describe a new species of *Amphictene* based on specimens collected from the coastal waters of Guangdong in the northern South China Sea.

## Material and methods

Specimens were collected during benthic ecology surveys conducted in Daya Bay and Honghai Bay of the northern South China Sea (Table 1). Sediment samples were collected using a 0.05 m^2^ or 0.1 m^2^ van Veen grab, and washed through a sieve with a 0.5 mm mesh size. Specimens were picked up from the sieve, fixed in 5% formalin and later transferred to 70% ethanol. Specimens were observed under a Carl Zeiss Stemi 2000-C dissecting microscope fitted with an AxioCam ICc 1 camera. Two paratypes were dehydrated using a Xiangyi CFD-10D freeze-dryer, gold coated with an EDT SC-150, and observed under a TESCAN CEGA 3 scanning electron microscope. Types are deposited in the following institutions: the Australian Museum, Sydney, Australia (AM); The Marine Biological Science Museum of the Chinese Academy of Sciences, Qingdao, China (MBM); The South China Sea Institute of Oceanology of the Chinese Academy of Sciences, Guangzhou, China (SCSMBC). The taxonomic terms defined by Hutchings and Peart (2002) are used for describing the species.

**Table 1.** Major morphological characters and collection information for the type specimens of *Amphictene alata* sp. n.

Catalogue number	Body length (mm)	Anterior body width (mm)	Number of cirri on cephalic veil	Pairs of paleae	Pairs of scaphal hooks	Number of lappets on opercular rim	Collection date (yyyy/mm/dd)	Location		Water depth (m)
								N(°)	E(°)	
**Holotype**										
MBM283388	27.9	4.6	15	9	26	22	2013/12/24	22.52333	115.09167	22.5
**Paratype**										
SCSMBC006677	29.4	5.3	11	10	26	24	2013/5/20	22.5880	114.5886	14.5
SCSMBC006678	36.1	3.9	13	8	29	22	2013/5/20	22.5880	114.5886	14.5
SCSMBC006679	34.5	4.7	13	8	37	24	2013/5/20	22.57611	114.68444	14.5
SCSMBC006680	38.8	5.8	15	9	39	24	2013/10/25	22.5880	114.6786	15.0
AMW.48292	33.0	4.0	14	10	32	21	2013/9/27	22.5914	114.5539	12.0
MBM283389	50.7	6.7	13	11	35	22	2013/9/27	22.55940	114.61190	20.0
MBM283390	48.8	6.9	n.r.	9	36	22	2013/9/27	22.5594	114.6119	20.0
MBM283391	30.8	4.0	13	9	34	20	2013/10/24	22.5914	114.5539	13.0
MBM283392	26.3	5.0	n.r.	9	33	n.r.	2013/8/28	22.55942	114.61192	16.0
SCSMBC006681	14.0	2.2	13	11	n.r.	20	2014/1/23	22.5914	114.5539	12.0
SCSMBC006682	10.2	2.4	n.r.	11	n.r.	20	2014/8/21	22.6247	114.6719	13.0
AMW.48293	25.0	4.0	16	10	26	25	2014/8/21	22.6083	114.7288	12.0
AMW.48294	16.0	2.8	16	9	25	22	2014/8/19	22.54550	114.87067	6.0
SCSMBC006683	45.2	5.8	16	10	32	22	2015/3/20	22.5930	114.5532	10.0

n.r.: character not recorded due to specimen damage.

The following abbreviations are used in figure legends: op, opercular palea; or, opercular rim; cv, cephalic veil; pp, peristomial palp; tc, tentacular cirrus; br, branchia; dll, dorsal lateral lobes (segment 3); c1, chaetiger 1 (segment 5); c4, chaetiger 4 (segment 8); c10, chaetiger 10 (segment 14); sc, scaphe; sh, scaphal hooks; al, anal lobe; s21, segment 21.

## Results

### FAMILY Pectinariidae de Quatrefages, 1866

#### GENUS Amphictene

Taxon classificationAnimaliaTerebellidaPectinariidae

Savigny in Lamarck, 1818

##### Type species.

*Amphitrite auricoma* Müller, 1776

#### Amphictene alata sp. n.

Taxon classificationAnimaliaTerebellidaPectinariidae

http://zoobank.org/0DE5D0F9-FCF9-4FDF-A300-786BC1A0F88C

**Figure 1.**
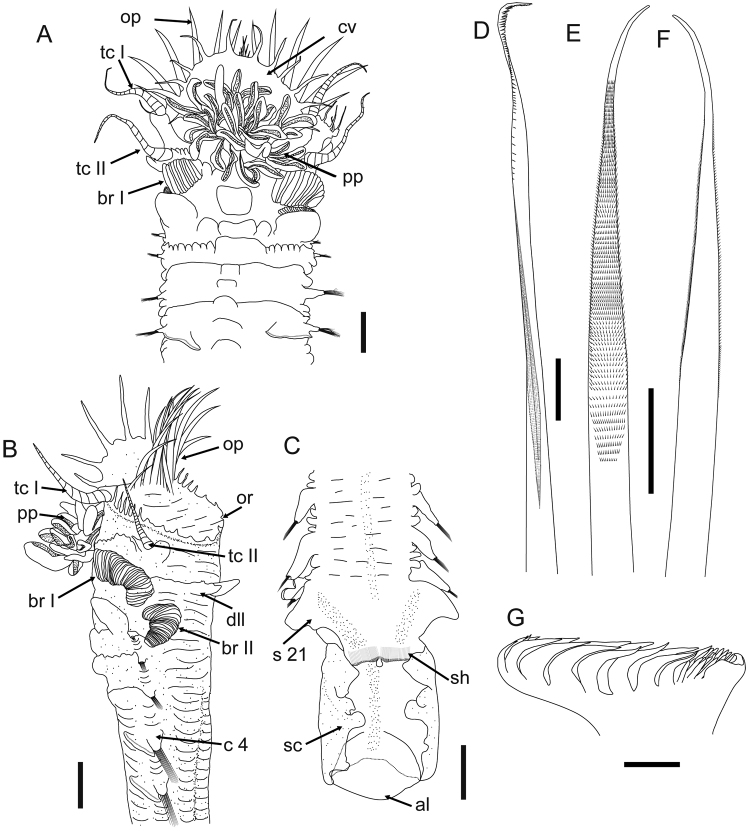
*Amphictene alata* sp. n., drawn from holotype MBM283388. **A** ventral view of anterior end **B** lateral view of anterior end **C** dorsal view of posterior end **D** ventral view of notochaeta 1 **E** ventral view of notochaeta, chaetiger 2 **F** dorsal view of notochaeta, chaetiger 2 **G** lateral view of neurochaeta uncinus. Scale bars: 1 mm (**A–C**); 50 μm (**D–F**); 5 μm (**G**). Abbreviations for morphological characters have been defined in Material and methods.

**Figure 2.**
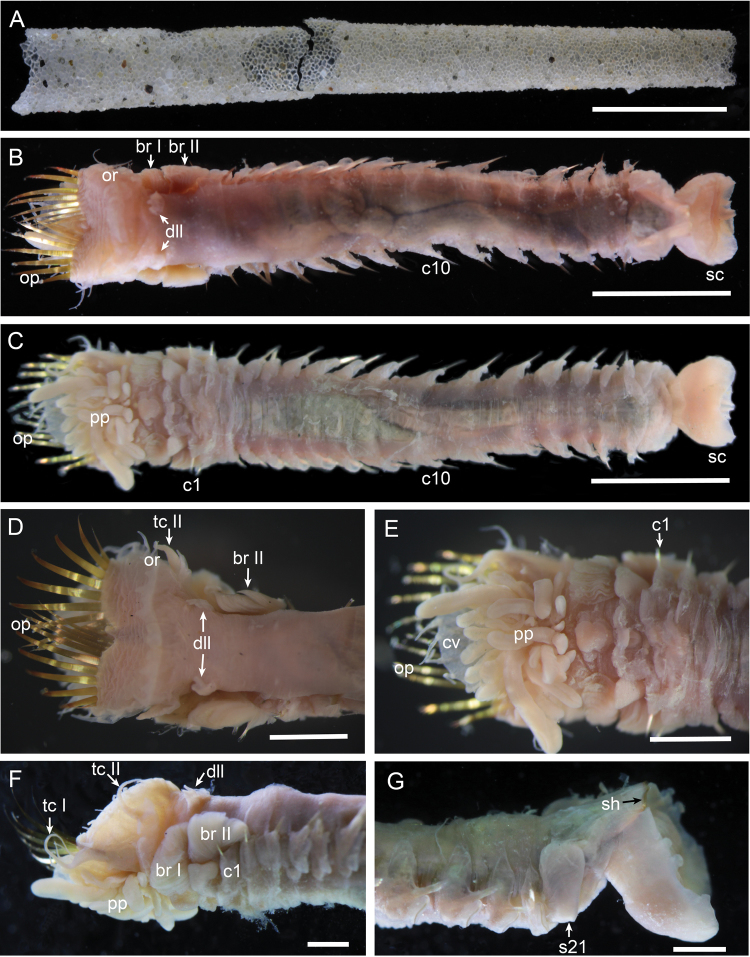
*Amphictene alata* sp. n. paratypes W.48293. **A** tube, broken in the middle **B** dorsal view of the whole worm **C** ventral view of the whole worm **D** dorsal view of anterior end **E** ventral view of anterior end **F** lateral view of anterior end **G** lateral view of posterior end. Scale bars: 1 cm (**A**); 5 mm (**B, C**); 2 mm (**D, E**); 1 mm (**F, G**). Abbreviations for morphological characters have been defined in Material and methods.

**Figure 3.**
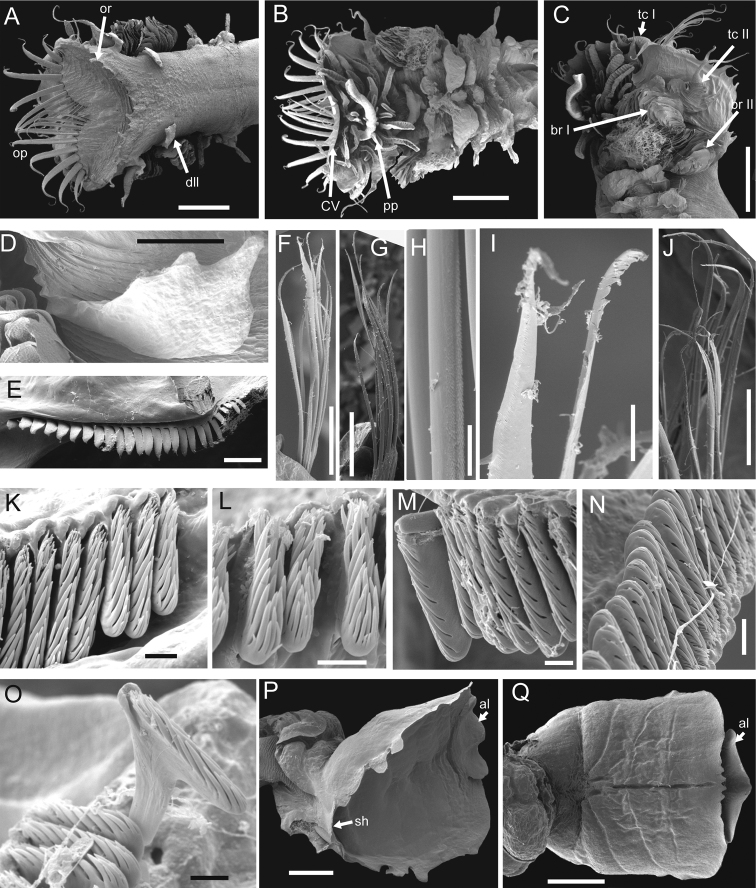
*Amphictene alata* sp. n. Scanning electron micrographs of paratype SCSMBC006683. **A** dorsal view of anterior end **B** ventral view of anterior end **C** lateral view of anterior end **D** left dorsal lateral lobe on segment 3 **E** some examples of the scaphal hooks **F, H, I** ventral view of notochaetae from chaetiger 2 **G** dorsal view of notochaetae from chaetiger 2 **J** ventral view of notochaetae from chaetiger 4 **K, L** and **M** ventral neurochaetae from chaetiger 4, 6 and 13, respectively **N** and **O** dorsal neurochaetae from chaetiger 5 and 16, respectively **P** dorsolateral view of scaphe **Q** ventral view of scaphe. Scale bars: 2 mm (**A–C**); 500 μm (**D**); 100 μm (**E, J**); 200 μm (**F, G**); 20 μm (H, I); 5 μm (**K–M, O**); 10 μm (**N**); 1 mm (**P, Q**). Abbreviations for morphological characters have been defined in Material and methods.

Figs 1 , 2 , 3 , Table 1

##### Material examined.

15 type specimens, all collected from the silt-clay bottom of Daya Bay and Honghai Bay, Guangdong Province, China at 6 to 22.5 m depth (Table 1). **Holotype**: MBM283388. **Paratypes**: MBM283389, MBM283390, MBM283391 and MBM283392, SCSMBC006677, SCSMBC006678, SCSMBC006679 (prepared for SEM), SCSMBC006680, SCSMBC006681, SCSMBC006682, SCSMBC006683 (prepared for SEM), AMW.48292, AMW.48293 and AMW.48294. Among them, SCSMBC006681 and SCSMBC006682 are incomplete with 14 anterior segments and 12 anterior segments, respectively. SCSMBC006679 and SCSMBC006683 are each broken into two fragments. Others specimens are complete.

##### Etymology.

The specific name *alata* is derived from *ala*, a Latin word for wing. It refers to the pair of wing-shaped dorsal lateral lobes on segment 3, a distinctive feature for this species.

##### Diagnosis.

Cephalic veil completely free from operculum forming dorsal semi-circle around numerous peristomial palps. Rim of cephalic veil with 11 to 16 long cirri. Dorsal operculum raised with 20–25 marginal cirri. Comb-like branchiae present on segments 3 and 4. A pair of dorsal lateral lobes present on segment 3. Chaetigers 1 to 3 (segments 5 to 7) with notopodia and notochaetae only. Chaetigers 4 to 16 (segments 8 to 16) biramous with notopodia, neuropodia, notochaetae and neurochaetae. Notochaetae winged capillaries. Neurochaetal uncini with major teeth arranged in two to three rows. Segment 21 with a pair of large lateral lobes but without chaetae. Scaphe distinctly separated from abdomen, with 26–37 pairs of short hooks with a slightly curved tip.

##### Description.

Preserved specimens pale cream to grey in colour. Body length of intact specimens including scaphe 16.0 to 50.7 mm, and greatest width at cephalic region 2.2 to 6.9 mm (Table 1). Cephalic veil completely free from operculum, forming dorsal semi-circular lobe surrounding numerous peristomial palps. Rim of cephalic veil with 11 to 16 long smooth cirri; cirri basally triangular, tapering to form terminal filament (Figs 1A, 2E, 3B). Doral opercular margin raised, crenulated with 8 to 13 triangular dorsal lappets and 5 to 6 pairs of short smooth lateral cirri (Figs 1B, 2D, 3A). Operculum with 8 to 11 pairs of golden paleae, fan-shaped on each side with ventral ones longer than lateral ones, curved dorsally with extended tips (Figs 1B, 2B, 3A).

Tentacular cirri subulate, annulate, tapering to apex (Figs 1A, B). First pair of tentacular cirri arises on ventral posterolateral margin of segment 1 (Figs 1B, 2F, 3C). Second pair of tentacular cirri present laterally on segment 2 with dorsal and ventral ear-shaped lobes at the base (Figs 1B, 2F, 3C). Segment 2 with an incised ventral ridge, forming glandular lobes (Fig. 1B). Segment 2 without anterodorsal lobe (Figs 1B, 2D, 3A).

Segment 3 with a large midventral lobe (Figs 1A, 2E, 3B), a pair of dorsolateral lobes with crenulated margin (Figs 1B, 2D, 2F, 3A, 3D), and a pair of lateral lamellate branchiae (Figs 1B, 2F, 3C).

Segment 4 with a small midventral lobe (Figs 1A, 2E, 3B), a pair of larger lateral ventral lobes (Figs 1A, 2E, 3B), and a pair of lateral lamellate branchiae (Figs 1B, 2F, 3C). Large subquadrate glandular lobes arise ventrally at base of branchiae (Fig. 1A). Segment 4 with smooth dorsum (Figs 1B, 2B, 3A).

Segments 5 to 7 (chaetigers 1 to 3) with notopodia and notochaetae only (Figs 1B, 2F); anteroventral lobe large and broad (Figs 1A, 2E, 3B). Segment 5 with a small midventral lobe, and a pair of huge lateral ventral lobes covering much of the venter. Segment 6 with a small midventral lobe, and a pair of larger lateral ventral lobes. Segment 7 with a raised ventral lobe running through the venter. Segments 8 to 20 (chaetigers 4 to 16) biramous with wedge-shaped notopodia and lobe-shaped neuropodia. Notopodia with two kinds of simple chaetae: one slender, with sub-distal serrations along one side (Figs 1D, 3F–I); the other stout, with finely hirsute surface on one side, tapering to an acute tip (Figs 1E, 1F, 3J). Neuropodia with uncini arranged in one row along raised ridge of the tori. Uncini with two or three longitudinal rows of teeth, each row with 7 to 9 major teeth (Figs 1G, 3K–O).

Segment 21 achaetous, with a pair of large lobes covering much of the lateral sides of the body (Figs 1C, 2G, 3P).

Scaphe formed by fusion of 5 posterior segments, distinctly separated from abdomen (Fig. 3Q). Scaphe ovoid, slightly convex dorsally, with two pairs of lappets on lateral margin (Figs 1C, 1D, 2G, 3P), and four small triangular lappets on distal margin (Fig. 3Q). Scaphal hooks 26 to 39 pairs, short, with a slightly bent tip (Fig. 3E). Anal tongue oval, with smooth margin extending beyond posterior scaphal margin (Figs 1C, 3P, 3Q).

Tube yellowish, conical, straight, composed of sand grains and shell fragments held together by cement (Fig. 2A).

##### Variation in morphological characters.

A number of morphological characters exhibit variations (Table 1). Specifically, the body length ranges from 10.2 to 50.7 mm, the widest width of anterior body ranges from 2.2 to 6.9 mm, the numbers of cephalic veil cirri range from 11 to 16, the pairs of paleae range from 8 to 11, the number of triangular lappets on the raised opercular rim varies from 20 to 25, and the number of pairs of scaphal hooks ranges from 25 to 37. The widest width of anterior body (BW) and body length (BL) are positively correlated: BL = 0.7469BW - 3.6038, R^2^ = 0.8252, *P* < 0.001, n = 15. Correlational analysis between BW, which is considered to be less affected by fixation than BL, and other quantitative parameters in Table 1 shows that BW had significant positive correlation only with the number of scaphal hooks (SH): SH = 2.3185BW + 20.213, R^2^ = 0.3419, *P* = 0.036, n = 13.

##### Type locality and distribution.

Currently only known from Daya Bay and Honghai Bay, Guangdong in the northern South China Sea.

## Discussion

*Amphictene alata* sp. n. can be distinguished from other currently recognized species in the genus by several features. First, it has a pair of dorsolateral lobes with crenulated margins on segment 3. None of the other species has dorsolateral lobes. Second, segment 21of *Amphictene alata* sp. n. has a pair of large lateral lobes but has no chaetae. Among the reported species of *Amphictene*, *Amphictene japonica* is also achaetous in segment 21, but it does not have large lateral lobes. Third, *Amphictene alata* sp. n. has more scaphal hooks (26-37 pairs) than other species of *Amphictene* (4 to 25 pairs). Forth, the scaphal morphology of *Amphictene alata* sp. n. is unique among *Amphictene* spp. in having 2 pairs of lateral lappets, four small triangular terminal lappets, and an oval anal plate with a smooth margin.

According to the generic diagnosis of Hutchings and Peart (2002), *Amphictene* has 17 chaetigers, with chaetigers 1 to 3 (segments 5 to 7) having notopodia and notochaetae only; chaetigers 4 to 16 (segments 8-20) biramous with notopodia, neuropodia, notochaetae and neurochaetae; and chaetiger 17 (segment 21) with notopodia and notochaetae only. However, *Amphictene alata* sp. n. has 16 chaetigers only, with the last chaetiger having notopodia, neuropodia, notochaetae and neurochaetae; and segment 21 is achaetous. *Amphictene japonica* also has 16 chaetigers, with the last chaetiger being biramous with notopodia, neuropodia, notochaetae and neurochaetae (Nilsson 1928; Okuda 1934; Nishi et al. 2014). *Amphictene helenae* has 15 chaetigers, followed by three achaetous segments anterior to the scaphe (García-Garza and de León-González 2014). Therefore the generic diagnosis should be amended to change that members of this genus can have 15 to 17 chaetigers.

## Supplementary Material

XML Treatment for Amphictene

XML Treatment for Amphictene alata
